# Transcriptional heterogeneity predicts and enables clonal selection in ageing haematopoiesis

**DOI:** 10.21203/rs.3.rs-10046174/v1

**Published:** 2026-06-25

**Authors:** Marco De Dominici, Kailiang Qian, Xunxuan Chen, Yang Liu, Qiuyang Zhang, James S Chavez, Xiaowen Chen, Travis Roeder, Hideyuki Oguro, Eric Pietras, James DeGregori, Sheng Li

**Affiliations:** 1. Department of Biochemistry and Molecular Genetics, University of Colorado Anschutz Medical Campus, Aurora, CO.; 2. Department of Cancer Biology, Keck School of Medicine, University of Southern California, Los Angeles, CA.; 3. Cancer Institute, Stanford University School of Medicine, Stanford, CA.; 4. Jackson Laboratory for Genome Medicine, Farmington, CT; 5. Department of Cell Biology, University of Connecticut School of Medicine, Farmington, CT.; 6. Division of Hematology, Department of Medicine, University of Colorado Anschutz Medical Campus, Aurora, CO.; 7. Department of Immunology and Microbiology, University of Colorado Anschutz Medical Campus, Aurora, CO, USA.; 8. Norris Comprehensive Cancer Center, University of Southern California, Los Angeles, CA.; 9. Department of Quantitative and Computational Biology, Dornsife College of Letters, Arts and Sciences, University of Southern California, Los Angeles, CA.

## Abstract

A general puzzle in stem-cell and ageing biology is why a few cellular clones come to dominate an ageing tissue while otherwise similar neighbours do not, a fate that the average transcriptional state of a cell predicts poorly. Here we ask whether the variability between sister cells of a clone, rather than their transcriptional state, is the property that predicts ageing-associated clonal selection, using the haematopoietic stem cell (HSC) as a tractable test case. We combine heritable lineage tracing with single-cell RNA sequencing across heterochronic and homochronic transplantation models to link early transcriptional states of individual HSC clones to their long-term functional output *in vivo*. To quantify transcriptional heterogeneity at clonal resolution, we developed a computational framework (scCloneVar) that estimates mean-adjusted gene expression variance and identifies differentially variable genes (DVG). We found that ageing increases transcriptional heterogeneity at both the cellular and clonal levels, reflected by elevated variability in gene expression programs that regulate stem cell activity. We observe polyclonal expansion of HSC independently of the age of the host or the donor mice; however, individual clones in heterochronic transplantations show reduced self-renewal and fitness compared to sister clones in homochronic transplantations, indicating better adaptation of HSC clones in age-matched microenvironments. Strikingly, transcriptional features measured prior to transplantation predict clonal self-renewal at later time points, with transcriptional variability, captured by DVG, providing predictive power beyond that captured by mean expression differences. DVG-associated programs are conserved across mouse and human HSC, are established by middle age, and are enriched in pathways relevant to clonal haematopoiesis and myeloid malignancy risk. Together, our findings support a model in which ageing expands transcriptional heterogeneity that tracks with subsequent clonal selection, rendering clonal fate partially predictable from early cellular states.

## One-sentence summary

Pre-existing transcriptional heterogeneity in HSC stratifies clonal self-renewal within aged niches, linking intrinsic ageing programs to clonal dominance.

Ageing profoundly remodels haematopoiesis, leading to reduced regenerative capacity, lineage skewing and an increased incidence of clonal haematopoiesis (CH) and haematopoietic malignancies^1–4^. At the cellular level, ageing is accompanied by transcriptional and epigenetic changes in haematopoietic stem cells (HSC), increased molecular variability^5^ and a progressive loss of clonal diversity. Human aged haematopoiesis becomes dominated by a limited number of self-renewing clones^6,7^, yet the principles governing which HSC expand with age, and why, remain poorly understood.

Single-cell technologies have provided detailed snapshots of age-associated HSC states, revealing altered transcriptional programs, epigenetic drift and increased heterogeneity. In parallel, lineage-tracing approaches have demonstrated that ageing is associated with clonal expansion and skewed contribution to blood production^8^. However, these advances have largely progressed along separate axes: single-cell studies lack longitudinal lineage resolution, whereas clonal analyses typically provide limited insight into the molecular states that precede clonal dominance. As a result, the relationship between HSC molecular state and long-term clonal behaviour during ageing remains unresolved.

A central unanswered question is whether clonal expansion in ageing arises from stochastic drift, *de novo* acquisition of advantageous states, or selective amplification of pre-existing HSC heterogeneity. Ageing affects both intrinsic HSC programs and extrinsic niche signals^9,10^, but disentangling their relative contributions to clonal evolution has been challenging. Without direct linkage between pre-existing transcriptional profiles and later clonal output, it is unclear whether ageing reshapes haematopoiesis by broadly degrading stem cell function or by selectively favouring a subset of HSC optimized for aged environments.

Recent lineage-tracing studies using endogenous mutations or epigenetic marks, including EPI-Clone^11^, have mapped age-associated clonal expansions in unperturbed haematopoiesis and identified epigenetic and inflammatory features within expanded clones. These studies established that ageing is associated with non-random clonal selection but the observations were limited to molecular feature of clones after *in vivo* expansion, precluding direct inference of how baseline transcriptional states influence future clonal fate. Complementary transplantation and barcoding studies manipulating donor and host age have further shown that aged HSC maintain clonal diversity similar to young HSC^12^. Still, the molecular mechanisms and predictors of clonal success during ageing have remained elusive.

A large body of literature supports the notion that ageing is associated with increased epigenetic and transcriptional heterogeneity, or noise, in multiple tissues, which is the basis for the “information theory of aging” (ITOA)^13^. ITOA posits that aging reflects a progressive loss of youthful epigenetic information that can, in principle, be retrieved through reprogramming. Regional epigenetic disorder in blood increases across animal species and scales with lifespan^14^. Ageing HSC show decreased gene-to-gene coordination^15^ and increased transcriptional noise^16^. Recent studies have focused on the effect of altered transcriptional states in determining HSC self-renewal and repopulation potential in ageing contexts, but transcriptional variability has not been examined as a factor determining stem cell behaviour.

Here, we address these gaps by combining heritable single-cell lineage tracing with transcriptomic profiling to prospectively link HSC molecular state to clonal fate across ageing contexts. By integrating clonal history, transcriptional state and functional output in heterochronic transplantation settings, we dissect how intrinsic heterogeneity and extrinsic ageing environments interact to shape HSC clonal evolution. This approach reveals ageing as a selective process that amplifies pre-existing HSC heterogeneity, providing a predictive framework for understanding stem cell ageing and age-associated clonal disorders.

## Single-cell lineage tracing links HSC transcriptional state to functional output *in vivo*

**Figure 1.**
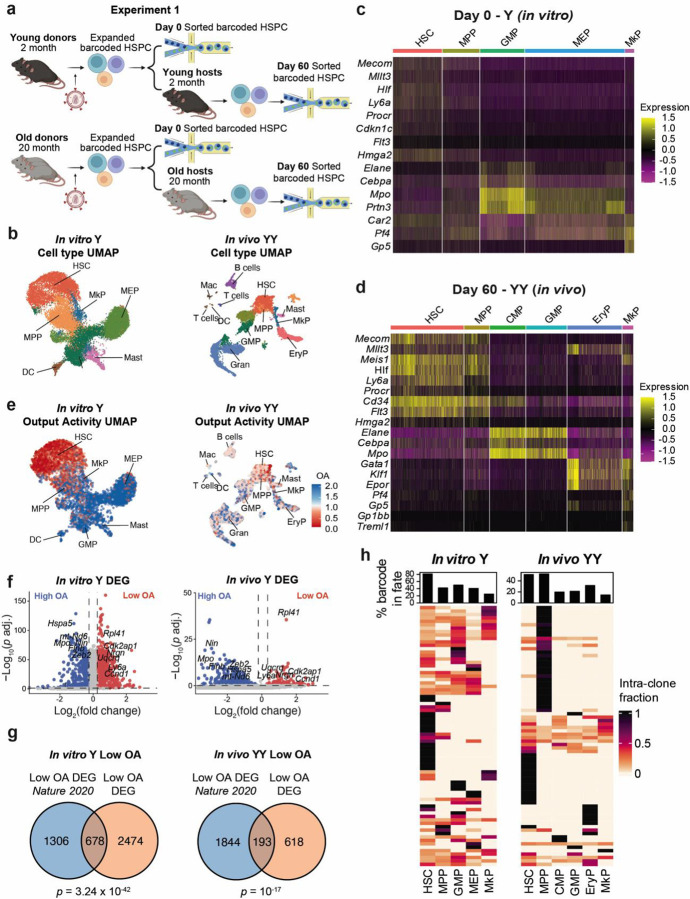
Simultaneous single-cell lineage and transcriptome sequencing maps functional HSC heterogeneity *in vivo*. **a,** Schematic of the CellTag barcoding experiment for young donor-young host transplantation experiment, with scRNA-seq performed at day 0 (*in vitro*) and day 60 (*in vivo*). **b,** UMAP of scRNA-seq profiles from HSPC (with added differentiated cells) in the young donor cells at day 0, *in vitro* (Y) or after transplantation into young hosts at day 60, *in vivo* (YY) from Experiment 1; left, *in vitro*; right, *in vivo*. Colours highlight groups identified from unsupervised clustering. Major progenitor cell populations are labelled. MPP, multipotent progenitor; CMP, common myeloid progenitor; GMP, granulocyte–monocyte progenitor; MkP, megakaryocyte progenitor; EryP, erythroid progenitor, DC, dendritic cell; Mac, macrophage; Gran, granulocyte; B-cell, B lymphocyte; T-cell, T lymphocyte; Mast, mast cell. HSC and progenitor cell annotations are based on **c-d**. **c,d,** Heatmap showing expression of known marker genes across single cells in each scRNA-seq cluster (using the Seurat package); **c**, Day 0, *in vitro;* **d**, Day 60, *in vivo*. **e,** Single-cell map that shows clonal HSC output activity (OA) values *in vitro and vivo*. CellTag barcodes are used to assign each cell to an HSC clone, and then all cells from each clone are coloured based on its calculated output activity. **f,** Differentially expressed gens (DEG) in high-output (left side) versus low-output (right side) HSC; left panel, *in vitro*; right panel, *in vivo*. Genes with an adjusted p < 0.05 (Benjamini–Hochberg correction) and fold change > 0.25 or < −0.25 are coloured. Selected shared genes between *in vitro* and *in vivo* are labelled. Red and blue dots represent low-output and high-output genes, respectively (*in vitro*: n = 671 and n = 375; *in vivo*: n = 112 and n = 357). g, Venn diagram showing overlap between low-OA reference genes reported previously^20^ and low-OA DEG from this study; left, *in vitro*; right, *in vivo*. Overlap significance was assessed using the hypergeometric test. **h**, State-bias heatmaps showing clones (rows) and clusters/states (columns), coloured by the intra-clonal fraction in each cluster/state (scale). Clones shown are all those detected in both Day 0 and Day 60. % barcode in fate indicates the percentage of clones (barcodes) that contribute at least one cell to each lineage.

A central question in HSC biology is how transcriptionally defined HSC states relate to functional output during haematopoiesis *in vivo*. To address this, we combined single-cell RNA sequencing (scRNA-seq) with heritable CellTag barcoding^17^ to simultaneously capture lineage relationships and transcriptional profiles of individual HSC following transplantation. Young donor HSC were barcoded *ex vivo* and transplanted into young recipient hosts, with scRNA-seq performed at the time of transplantation (day 0, *in vitro*, **Experiment 1, Extended Data Table 2 and 3**) and after long-term engraftment (day 60, *in vivo*) (Fig. 1a, **Experiment 1, Extended Data Table 4**). To relate transcriptional state to functional output (i.e. production of progenitor and differentiated cells), CellTag barcodes were used to reconstruct clonal relationships and quantify the differentiation output of individual HSC clones (**Extended Data Fig. 1a-d**). To minimize perturbation of the host bone marrow microenvironment, we employed a low-dose busulfan conditioning regimen that selectively depletes host lineage^NEG^, Sca-1^+^, c-Kit^+^/haematopoietic stem and progenitor cells (HSPC) cells, thereby preserving overall bone marrow cellularity with only minimal, transient inflammation that resolves by the time of transplantation^18^.

Initially focusing on the young contexts, unsupervised clustering of scRNA-seq profiles from HSPC and mature haematopoietic cells revealed distinct transcriptional states at both time points. As expected, uniform manifold approximation and projection (UMAP) embedding showed clear separation of clusters *in vitro* at day 0 and *in vivo* at day 60, indicating that HSPC occupy discrete transcriptional states that are reshaped following transplantation and *in vivo* differentiation (Fig. 1b). Cluster identities were assigned based on the expression of canonical HSC and progenitor markers (Fig. 1c,d, **Extended Data Fig. 1e,f**), allowing annotation of major populations, including HSC, multipotent progenitors (MPP), lineage-primed progenitors and committed myeloid, lymphoid and erythroid progenitors.

We quantified an output activity (OA) metric^19^ that captures the extent to which each HSC clone contributes to downstream progenitor populations. The higher the clonal HSC self-renewal, the lower the OA. This analysis revealed substantial heterogeneity in clonal output, with some HSC clones exhibiting low output (i.e., high self-renewal) and remaining largely restricted to stem or early progenitor states, whereas others showed high output with broad contribution to multiple downstream lineages. Importantly, this functional heterogeneity was evident both *in vitro* and *in vivo* (Fig. 1e).

We next asked whether low- and high-output HSC could be distinguished transcriptionally. Differential gene expression analysis comparing low-output and high-output HSC identified distinct gene expression programs associated with functional output in both settings (Fig. 1f). *In vitro*, high-output HSC displayed elevated expression of genes associated with proliferation and lineage priming, whereas low-output HSC were enriched for genes previously linked to stemness and quiescence. A comparable transcriptional separation was observed *in vivo*, indicating that functional output correlates with context-dependent transcriptional programs following transplantation.

To assess the relationship between these signatures and previously described low-output HSC markers, we compared our differentially expressed genes with a published low-output HSC gene set^20^, using all genes. Significant overlap was observed in both the *in vitro* and *in vivo* comparisons (*p* < 0.005, hypergeometric test), supporting the robustness of the identified transcriptional programs and their relevance to HSC functional heterogeneity (Fig. 1g).

Finally, we examined how individual clones distributed across transcriptional states over time. State-bias heatmaps revealed clone-specific patterns of occupancy across clusters, with some clones preferentially contributing to restricted states and others spanning multiple progenitor compartments (Fig. 1h). Together, these results demonstrate that combining single-cell lineage tracing with transcriptomic profiling enables direct linkage of HSC functional output to transcriptional state.

## Conserved transcriptional programs of HSC clonal output across young and aged donor and host contexts

**Figure 2.**
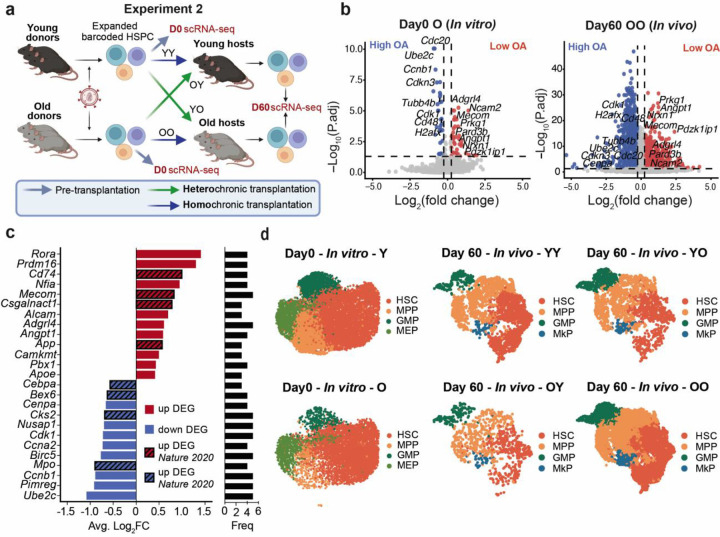
Conserved transcriptional programs of HSC clonal output across young and aged donor and host contexts. **a,** Schematic of the CellTag barcoding workflow for the young/old donor × young/old host transplantation experiment, with scRNA-seq performed *in vitro* at day 0 (D0) and *in vivo* at day 60 (D60). **YY**, young donors transplanted into young hosts; **YO**, young donors into old hosts; **OY**, old donors into young hosts; **OO**, old donors into old hosts. **b,** Genes differentially expressed in high-output (left side) versus low-output (right side) HSC; left panel, in old donor at day 0 (D0-O) *in vitro*; right panel, in old host at day 60 (D60-OO) *in vivo*. Genes with an adjusted *p* < 0.05 (Benjamini–Hochberg-corrected MAST test) and fold change of >0.25 and < −0.25 are coloured. Selected shared genes between *in vitro* and *in vivo* are labelled. Red and blue dots represent low-output and high-output genes, respectively (*in vitro*: n = 46 and n = 33; *in vivo*: n = 457 and n = 610). **c,** left, Average log_2_ fold change of the most frequently identified OA DEG across six samples (D0: O, Y; D60: OO, OY, YO, YY), coloured bars indicate overlap with previously described OA markers^20^; right, Frequency of each gene being identified as a DEG across six samples. **d,** Integrated contour UMAP of young/old donor × young/old host transplantation cells (all clones combined) at day 0 (D0) and day 60 (D60), coloured by cluster group.

We next asked how donor age and host age individually influence HSC clonal behaviour and whether ageing alters the emergence of expanded, high-output clones. To address this, we performed CellTag barcoding followed by homochronic and heterochronic transplantation across all four combinations of young and old donors and hosts, with scRNA-seq profiling at day 0 *in vitro* (**Experiment 2, Extended Data Table 5 and 6**) and day 60 *in vivo* (Fig. 2a, **Experiment 2, Extended Data Table 7**) obtaining comparable numbers of labelled HSC and clones (**Extended Data Table 8**). CellTag representation per clone was consistent across transplantation conditions in both *in vitro* and *in vivo* samples, indicating stable barcode recovery and clonal representation (**Extended Data Fig. 2a**). Unsupervised clustering of *in vivo* scRNA-seq profiles identified canonical HSC and progenitor populations across all transplantation conditions, as defined by established marker gene expression (**Extended Data Fig. 2b,c**).

To determine whether ageing-associated transcriptional programs were linked to functional output, we compared gene expression between low-output and high-output HSC. Differential expression analysis identified gene signatures associated with low clonal output activity (low-OA, i.e., high self-renewal) in old donors at day 0 and in donor-derived cells transplanted into old hosts at day 60 (Fig. 2b). We observed a significant overlap of differentially expressed genes (DEG) identified in this study with those previously reported in low-OA young HSC^20^ (Fig. 2c, **Extended Data Table 9**), suggesting that both *in vitro* and *in vivo* clonal tracing capture transcriptional determinants of HSC functional output. Clonal output activity and associated transcriptional differences across HSPC clusters were consistent with patterns observed across conditions, including variation in output activity (lowest in HSC; **Extended Data Fig. 2d**) and differential expression based on output activity and age (**Extended Data Fig. 2e,f**). We observed significant overlap of low clonal output activity DEG in donors at day 0 and in hosts at day 60 (**Extended Data Table 9**), suggesting that transcriptional programs related to clonal self-renewal persisted after homochronic transplantation.

Young and old donor cells exhibited similar transcriptional states *in vitro* and were able to reconstitute major haematopoietic populations *in vivo* in all conditions but with noticeable differences in frequencies, particularly in microenvironments mismatched for age (Fig. 2d).

To characterize the overall clonal architecture across conditions, we compared clonal size distributions in homo- and hetero-chronic transplantation settings. We observed no detectable differences in overall clonal size distributions between young and old hosts, nor between matched and mismatched donor-recipient age combinations (Y versus O, YY versus YO and OO versus OY: *p* = 0.37, *p* = 1, *p* = 0.19, two-tailed Wilcoxon rank sum test) (**Extended Data Fig. 3a**). Moreover, even among the top-ranked clones with the highest HSC, MPP or myeloid output, clonal sizes were statistically indistinguishable between young and aged environments (**Extended Data Fig. 3b,c)** (*p* > 0.2, Wilcoxon rank sum test). The observed clonal diversity reflects the initial labeling landscape established by lentiviral transduction, with subsequent expansions and losses over the two-month study period. Thus, within the timespan of our studies (~2 months), age does not appear to impact CellTag-mediated generation of clonal diversity.

## Ageing increases transcriptional variability and clonal heterogeneity in HSC

**Figure 3.**
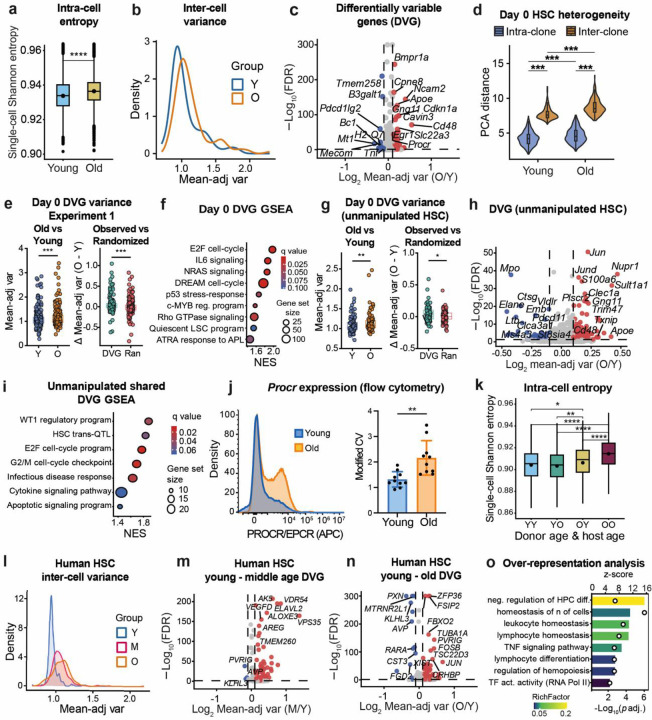
Ageing increases transcriptional variability and state heterogeneity in HSC. **a,** Quantification of intra-cellular HSC entropy between young and old HSC *in vitro* at day 0 **** unpaired t-test p < 0.0001 from Experiment **2**. **b**, Mean-adjust variance of expressed genes, comparing inter-cellular heterogeneity between young and old HSC in vitro at day 0 from Experiment **2**. **c**, Volcano plot of age-associated differentially variable genes (DVG) in HSC from Experiment **2**; Genes with an adjusted *p* < 0.05 (Bown-Forsythe-test with Benjamini–Hochberg correction) and fold change of >0.25 are coloured. Selected genes are labelled. Red and blue dots represent high variability in old HSC and young HSC, respectively (n = 117 and n = 20). **d**, Intra- and inter-clonal heterogeneity based on PCA dimension reductions (N = 30), comparing young (n = 1039) and old (n = 895) HSC clones at D0 from Experiment **2**. *** Wilcoxon rank-sum test p < 0.001. **e**, left, heterogeneity of ageing-associated DVG identified in Experiment 2 was evaluated in HSC gene expression from Experiment 1; right. The difference in mean-adjusted variance between young and old groups was compared for ageing-associated DVG and randomly selected genes. **f**, GSEA for ageing-associated DVG identified *in vitro* (Day 0) from Experiment 2. Dot size indicates gene set size, and colour represents q-value. NES, normalized enrichment score. **g**, left, Heterogeneity of ageing-associated DVG in Experiment 2 was independently evaluated in HSC from the unmanipulated experiment; right, Differences in mean-adjusted variance between young and old groups for ageing-associated DVG were compared with those of randomly selected genes (right) Quantification of mean-adjusted variance between young and old donors (left) and the absolute difference in mean-adjusted variance between the two groups (right) in unmanipulated HSC. Observed values from the top 100 DVG are compared with those from randomly selected genes. **h**, Volcano plot of age-associated differentially variable genes (DVG) in unmanipulated HSC. Selected genes are labelled. Red and blue dots represent genes with high variability in old and young HSC, respectively (n = 114 and n = 60). **i**, GSEA of shared ageing-up DVG between (**c**) and (**h**). **j**, Expression distribution of PROCR by flow cytometry control-vector transduced and *in vitro* expanded young or old HSC from 10 individual mice. **k**, Quantification of intra-cellular entropy in young/old donor × young/old host HSC from Experiment 2. Statistical significance was assessed using unpaired t-test. **l**, Mean-adjust variance of expressed genes, comparing inter-cellular heterogeneity between young (Y, 25–32 y, n = 3), middle-aged (M, 35–53 y, n = 3) and old (O, 62–77 y, n = 3) HSC from GSE189161. Pairwise Wilcoxon tests reveal age-dependent heterogeneity, with young HSC showed significantly lower mean-adjusted variance than middle-aged (p < 1.6 × 10^−30^) and old HSC (p < 5.1 × 10^−25^), and middle-aged HSC also exhibited lower mean-adjusted variance than old HSCs (p < 0.03). **m**, Volcano plot of young to middle age DVG in HSC; genes with an adjusted *p* < 0.05 (Bown-Forsythe-test with Benjamini–Hochberg correction) and absolute fold change of >0.25 are coloured. Selected genes are labelled. Red and blue dots represent high variability in middle aged HSC or young HSC, respectively (n = 114 and n = 3). **n**, same as **m** but representing DVG in old compared to young HSC (n = 105, n = 13). **o**, Over-representation analysis (ORA) of human DVG showing enriched biological pathways; dot indicates significance (−Log_10_ adjusted *p* value) and bar colour represents enrichment magnitude (RichFactor).

We next asked whether ageing alters not only mean transcriptional state, but also variability within transcriptionally defined HSC states. To quantify cell-to-cell variability, we measured intracellular transcriptional entropy^22^, a measure of a cell’s gene expression disorder or uncertainty, in young and old HSC after transplantation into aged-matched mice. Old HSC exhibited significantly higher entropy than young HSC (**Experiment 2, Fig. 3a**), indicating increased transcriptional heterogeneity with ageing. Consistent clonal representation across *in vitro* and *in vivo* conditions in independent experiments further supports robustness of clone detection (**Extended Data Fig. 4a**).

To quantify transcriptional variability at clonal resolution, we developed a computational framework (scCloneVar) that estimates mean-adjusted gene expression variance and identifies differentially variable genes (DVG), enabling separation of variability-driven signals from conventional differential expression. Using this approach, we observed a global increase in transcriptional variability across HSC from old relative to young mice (Fig. 3b, *p* < 0.0001, Wilcoxon rank sum test), accompanied by a set of DVG exhibiting significantly higher variance in old compared to young HSC (Fig. 3c). Importantly, increased heterogeneity was evident both within and between clones, with elevated intra-clonal and inter-clonal transcriptional divergence in aged donors (Fig. 3d), indicating enhanced rates of diversification of transcriptional states at older ages.

The same DVG observed in Experiment 2 (Fig. 3c) exhibited increased variability in old HSC from Experiment 1, while no increase in variability with age was observed for randomly selected gene sets (Fig. 3e). Consistent with this, transcriptional programs associated with functional output were reproducible across independent experiments (**Extended Data Fig. 4b**).

We performed gene set enrichment analysis (GSEA) on age-associated DVG from Experiment 1 at Day 0 and we observed significant enrichment in signatures relevant for the regulation of HSC self-renewal and quiescence (E2F cell cycle, DREAM cell cycle^23^, c-MYB regulatory program, Rho GTPase signalling^24^, Quiescent LSC program) and pathways altered in leukaemia (c-MYB regulatory protein^25^, NRAS signalling and ATRA response to APL) (Fig. 3f, **Extended Data Table 10**). These results indicate that transcriptional variability represents a structured and regulated property of HSC state, rather than stochastic noise, which could derive from age-dependent alterations in the level of engagement of specific transcriptional regulators, or possibly originate from differential selective evolutionary pressures in the aging bone marrow microenvironment.

We extended our analysis to unmanipulated, non-transplanted bone marrow cells from old or young donor mice (**Extended Data Fig. 4c**). Confirming the robustness of our data, our previously defined DVG set (Fig. 3c) exhibited increased variability in unmanipulated HSC from old mice relative to young, which was not observed in randomly selected control genes (Fig. 3g). Unmanipulated HSC show additional ageing-associated DVG (Fig. 3h). GSEA on shared DVG between the unmanipulated dataset and Experiment 2 also revealed enrichment for pathways involved in the regulation of HSC quiescence/self-renewal and normal and malignant haematopoiesis (Fig. 3i). These findings demonstrate that ageing-associated heterogeneity is neither random nor an artifact of experimental manipulation. Rather, DVG capture biologically meaningful heterogeneity in gene programs rather than generic increases in transcriptional noise (**Extended Data Table 11**).

Among DVG, *Procr* showed consistently altered expression distributions between young and old HSC across CellTag experiments and unmanipulated samples (**Extended Data Fig. 4d**). We validated this variability at the protein level via flow cytometry of young and old HSC transduced with a control lentiviral vector and expanded in similar conditions (Fig. 3j). This is notable as PROCR/EPCR has been previously described as modulator of HSC homing and retention in the bone marrow^26^ and a marker for repopulating HSC^27^, thus suggesting that variation in *Procr* expression may relate to changes in the functional properties of aged HSC. We also analysed the transcriptional heterogeneity of HSC in vivo at day 60 and we observed that while an aged microenvironment did not increase the transcriptional variability of young HSC, old HSC showed reduced single-cell Shannon entropy in young compared to old hosts (Fig. 3k), possibly due to a direct effect of the microenvironment on transcriptional regulation or because of differential clonal selection.

We next sought to validate our observation in human HSC. We analysed a dataset of HSC from 3 young (18–20 years old) and 5 old (>65 years old) donors^28^. Conservation of transcriptional programs and marker expression across species is supported by human bone marrow datasets (**Extended Data Fig. 4e**). Old HSC showed a significant increase in mean-adjusted variance compared to young (**Extended Data Fig. 4f**).

To determine when this ageing-associated variability signature is established, we analysed an independent human lifespan HSC dataset^29^ spanning young (25–32 y; n = 3), middle-aged (35–53 y; n = 3) and elderly (62–77 y; n = 3). Mean-adjusted variance increased progressively with age (pairwise Wilcoxon: young vs middle, *p* < 1.6 × 10^−30^; young vs old, *p* < 5.1 × 10^−25^; middle vs old, *p* < 0.03), with middle-age HSC already showing elevated variance relative to young-adult HSC (Fig. 3l). These data indicate that transcriptional heterogeneity in HSC begins to rise by middle age and continues to accumulate into late life. Specific sets of genes showed increased variability in middle aged compared to young HSC (Fig. 3m) and young compared to old HSC (Fig. 3n). Moreover, a significant set of genes show continuous increase in variance from middle aged to old (**Extended Data Fig. 4g, Extended Data Table 13**). Increase variability in DVG does not require increase in mean expression of the gene (**Extended Data Fig. 4h**). Notably and consistently with mouse data, human ageing-up DVG are not random but are enriched for transcriptional programs relevant to HSC stemness (with the top pathway being related to HSC differentiation), homeostasis and response to inflammation (Fig. 3o, **Extended Data Table 13–14**).

## Heterochronic transplantation separates intrinsic and extrinsic determinants of HSC clonal behaviour

**Figure 4.**
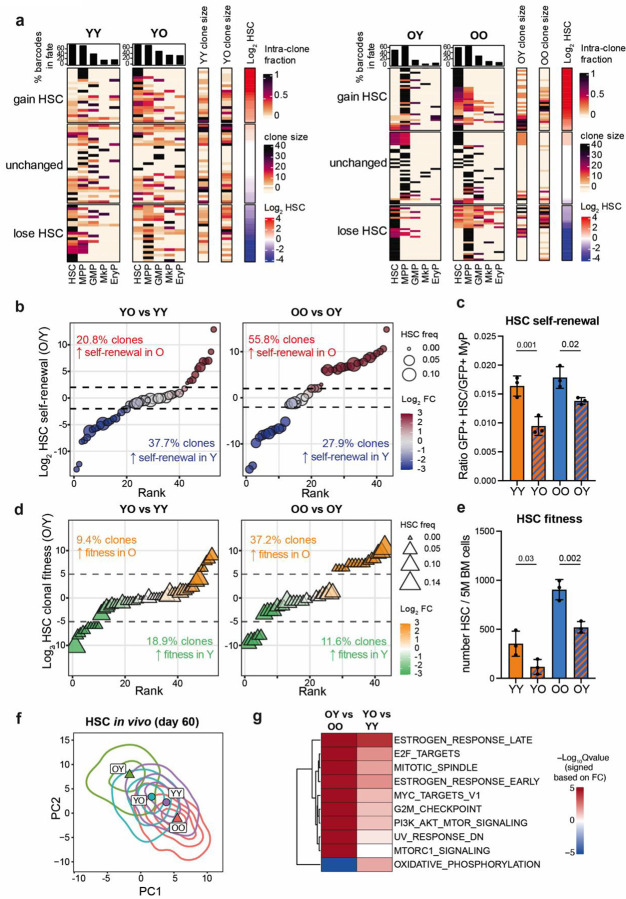
Heterochronic transplantation dissects intrinsic and extrinsic factors of HSC clonal behaviours. **a,** State-bias heatmap of sister clones observed in both young and old hosts at Day 60. Rows are aligned (same clone in each line) across all columns and ranked according to HSC Log_2_ fold-change. Additional columns show, for every clone, clonal proportion (size) in each culture and fold-change in HSC bias (old host over young host); left: young donor, n = 66; right: old donor: n = 100. **b,** Waterfall plot showing relative HSC self-renewal bias in old hosts compared with young hosts; left, young donors; right, old donors. The scale indicates changes in HSC self-renewal, from red (higher in old hosts) to blue (higher in young hosts). Bubble size denotes the HSC frequency in the condition where it was highest. A Log_2_ FC cutoff of −2 was used to define high- and low–self-renewal clones. Statistical significance was assessed using the Pearson Chi-square test, with *p* < 0.005 considered statistically significant. **c,** Bulk flow cytometry analysis showing HSC-to-myeloid progenitor ratios in host bone marrow across transplantation conditions, showing reduced self-renewal in heterochronic settings. **d,** Waterfall plot showing relative HSC clonal fitness bias in old host with respect to young host conditions; left: young donor; right: old donor. The scale indicates HSC clonal fitness change, from yellow (higher in old host) to green (higher in young host). Triangle size indicates the HSC frequency in the condition where it was highest. A Log_2_ FC cutoff of −5 was used to define high- and low–clonal fitness index clones. Statistical significance was assessed using the Pearson Chi-square test, with *p* < 0.005 considered statistically significant. **e,** Bulk flow cytometry analysis of bone marrow engraftment showing preferential expansion of young HSC in young hosts and old HSC in aged hosts. **f**, Principal component analysis (PCA) plot showing contours and density peaks in PC1-PC2 space for the HSC transplanted in different donor-host combination isolated 60 days after transplantation. **g**, Single-cell pathway analysis (SCPA) showing top 10 transcriptional pathways (by fold change, FC) differentially regulated in heterochronic transplanted HSC (OY or YO) compared to homochronic transplanted HSC (OO or YY) at day 60. q value scale relates to the number of genes changed, and the q value sign indicates the direction of change (positive means enriched in heterochronic transplanted HSC).

We next tested to what extent intrinsic or extrinsic aging alterations translate into differential clonal selection. Specifically, we directly compared properties of sister clones engrafting in both young and old hosts. State-bias heatmaps visualize, for each clone, the relative distribution of its progeny across transcriptionally defined cell states, thereby capturing clone-specific lineage and self-renewal biases^19^. State-bias heatmaps revealed that sister clones undergo systematic shifts in lineage output and HSC biases depending on host age, despite sharing a common clonal origin (Fig. 4a).

We categorized each clone by whether it gained or lost HSC self-renewal (HSC to non-HSC fraction within clones), or remained unchanged, in old hosts relative to young. We then assessed whether clonal divergence in HSC self-renewal was greater across age-mismatched environments than within age-matched replicates. Waterfall analysis revealed systematic, age-dependent shifts in HSC self-renewal (Fig. 4b, **Extended Data Table 15–16**). Notably, clones preferentially increased self-renewal in age-matched environments, indicating a selective advantage when donor and host ages are aligned. For HSC clones from old donor cells, we observe a larger proportion of clones showing strong self-renewal gains in old hosts (55.8%), with fewer clones preferentially maintained in young hosts (27.9%) (Fig. 4b), while young donor cells exhibit a higher self-renewal to young (37.7%) vs old (20.8%) hosts. The heterochronic transplantations showed a higher self-renewal than homochronic transplantation (*p* < 0.005, Pearson Chi-square test). A similar pattern was observable by bulk immunophenotypic population analysis that showed reduced HSC to myeloid progenitor ratio, indicative of lower self-renewal, in heterochronic than in homochronic transplantations (Fig. 4c).

In parallel, analysis of overall clonal fitness (relative expansion of each clone after transplantation) revealed host-dependent shifts in clonal dominance (Fig. 4d). Notably, while 37.2% of clones from old donors exhibit high fitness in old recipients (OO), only 11.6% exhibit high fitness in young recipients (OY) with an opposite trend for young donor cells which showed increased clonal fitness in young (18.9 %) versus old host (9.4%). Clonal self-renewal and fitness exhibited consistent relationships across shared clones, indicating that functional outputs are coordinated rather than independently varying across environments (**Extended Data Fig. 5a**). In agreement with these patterns, bulk population analysis by flow cytometry shows higher engraftment in homochronic compared to heterochronic conditions (Fig. 4e), indicating that clonal fitness is maximized in age-matched donor-host contexts. It is important to note that similar engraftment levels are obtained from old and young donors when comparable fractions of bone marrow HSC are transplanted, given that with ageing the HSC compartment increases in size but the engraftment potential of HSC is reduced on a per-cell basis (**Extended Data Fig. 5b**).

To gain insight into how the different donor-host age combinations shape the transcriptional state of transplanted HSC, we performed principal component analysis (PCA). Heterochronic samples (YO and OY) diverge from the aging axis of homochronic samples (YY to OO) with a leftward shift along PC1, suggesting activation of transcriptional programs specific for age-mismatched transplantations. Moreover, young HSC across microenvironments (YO vs YY) appeared less divergent than old donor HSC (OY vs OO, Fig. 4f). Replicate-level pseudo-bulk PERMANOVA confirmed a significant donor × host-age interaction (R^2^ = 0.28, P = 1.0 × 10^−4^; **Extended Data Fig. 5c**), suggesting asymmetric, donor-age-dependent responses. Comparing heterochronic samples to homochronic samples by single-cell pathway analysis (SCPA), multiple top differentially activated pathways were concordantly upregulated in both heterochronic comparisons relative to their homochronic counterparts, with stronger enrichment in old donor cells (OY vs OO greater than YO vs. YY, *p* = 0.0059, paired Wilcoxon test, Fig. 4g). Together, these observations suggest that heterochronic transplantation does not simply shift the transcriptome towards a host-age-matched state, but engages a shared set of pathways largely independent donor or host age. The greater transcriptional divergence in OY vs OO compared with YO vs YY further suggests that the elevated baseline transcriptional heterogeneity of aged HSC may enable broader adaptability to mismatched microenvironments.

## Pre-existing intra-clonal heterogeneity in old donors predicts adaptation to ageing environments

**Figure 5.**
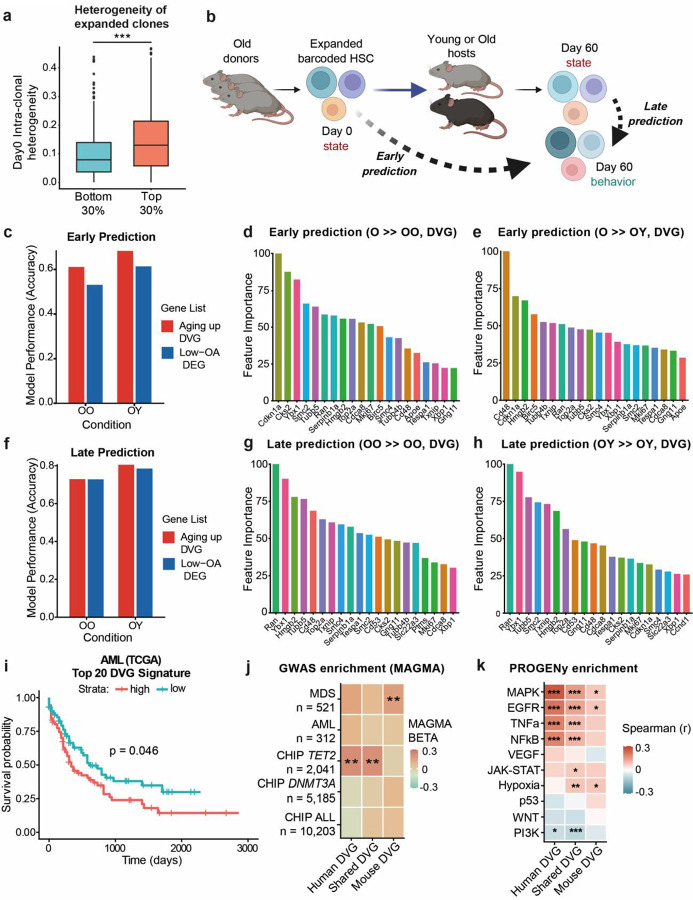
Pre-existing HSC intra-clonal heterogeneity has predictive value for self-renewal *in vivo* and correlated with risk of haematological malignancies. **a,** Relationship between pre-existing intra-clonal heterogeneity (D0) and *in vivo* clone size (D60) by HSPC frequency for old HSC clones. Clones were stratified into bottom 30% and top 30% based on D60 HSPC clone size. A significant difference in intra-clonal heterogeneity was observed between groups in the observed data (Wilcoxon Rank Sum test, *p* < 0.001). **b,** Schematic of machine learning models used to predict clonal fate from the transcriptional state of early progenitors *in vitro* (Day 0, early prediction) or *in vivo* (Day 60, late prediction). **c,** Performance (macro F1 score) of 5-fold cross-validation random forest models for early prediction of self-renewal status using gene expression profiles from *in vitro* (Day 0), based on DEG and DVG gene sets (>> indicates input to output prediction). **d,** Feature importance (the top 20 genes in DVG) in built early prediction model for predicting self-renewal in OO. **e,** Feature importance (the top 20 genes in DVG) in built early prediction model for predicting self-renewal in OY. **f,** Performance (macro F1 score) of 5-cross validation random forest model for late prediction for self-renewal status using gene expressions *in vivo* (Day 60), based on DEG and DVG gene sets. **g,** Feature importance (the top 20 genes in DVG) in built late prediction model for predicting self-renewal in OO. **h,** Feature importance (the top 20 genes in DVG) in built late prediction model for predicting self-renewal in OY. **i,** Survival analysis in the TCGA LAML dataset using individual genes selected from the top 20 feature-importance genes derived from the DVG gene set identified using a log2 fold-change cutoff of 0 for late prediction in old donors and old hosts. **j,** GWAS MAGMA enrichment analysis of DVG-associated genes. Heatmap showed the enrichment of human, mouse and shared DVG gene sets in ageing-related GWAS traits; colour indicates MAGMA beta values, and asterisks denote statistical significance. **k,** PROGENy pathway enrichment analysis of DVG gene sets (human, mouse and shared) in human GSE189161 data. DVG identified (FC > 0.05, *p* < 0.05) in at least two experiments (human or mouse) were used. Colour represents Spearman correlation coefficients, and asterisks indicate statistical significance.

We next asked whether transcriptional heterogeneity contributes to clonal self-renewal. Clones exhibiting higher intra-clonal heterogeneity at day 0 (pre-transplantation) showed increased self-renewal following transplantation, indicating that early transcriptional diversification is associated with subsequent clonal fitness (Fig. 5a). This relationship was not observed under random assignment, confirming that it reflects structured biological variation rather than stochastic effects (**Extended Data Fig. 6a**). These observations again suggest that variability within transcriptional states is not merely noise, but instead may encode latent differences among cells that influence clonal behaviour. To directly test whether early transcriptional states contain predictive information about future clonal outcomes, we trained models using gene expression profiles at day 0 (“early prediction”) and day 60 (“late prediction”) (Fig. 5b). Clonal fate was defined by the distribution of progeny across transcriptionally defined cell states, linking early transcriptional variability to lineage-resolved functional output.

DVG-based random forest models predicted clonal self-renewal with comparable or superior performance to DEG-based models from old donor HSC (Fig. 5c, **Extended Data Table 17**). Feature importance revealed an almost complete overlap between predictors in homochronic (OO) and heterochronic transplantations (OY), albeit with some difference in rank orders (Fig. 5d,e), indicating that transcriptional variability encodes information not captured by mean expression. These results suggest that variability reflects a distinct axis of HSC state that contributes to clonal fate beyond mean transcriptional programs. Model performance and feature selection were robust across configurations **(Extended Data Fig. 6b,c).** Prediction accuracy improved using day 60 transcriptional profiles (Fig. 5f), consistent with progressive coupling between transcriptional state and realized clonal outcome. As in early models, DVG-based predictors showed substantial overlap (Fig. 5g,h), indicating that variability contributes complementary predictive signals across time. Feature importance in late models was similarly stable **(Extended Data Fig. 6d).**

We next assessed the clinical relevance of these programs. While the preceding analyses focused on the DVG gene set identified using a more stringent log2 fold-change cutoff (log2FC = 0.1), survival analyses were performed using a broader DVG gene set identified using a log2 fold-change cutoff of 0. A DVG-derived gene signature and individual high-importance DVGs derived from the feature selection analysis of late-prediction models were also prognostic in The Cancer Genome Atlas (TCGA) AML cohort (Fig. 5i and **Extended Data Fig. 6e-h**), with higher expression associated with worse survival, underscoring the functional and clinical relevance of ageing-associated HSC clonal programs. To further assess the biological relevance of ageing DVG in modulating the risk of myeloid malignancies in humans, we assessed the overlap between human, mouse or shared set of ageing DVG to genes with demonstrated relevance to myeloid malignancies by genome-wide association studies (GWAS) by performing Multi-marker Analysis of GenoMic Annotation (MAGMA) analysis^30^. DVG-associated genes showed significant enrichment in *TET2* CHIP^31^ and MDS^32^ loci (Fig. 5j), with consistent enrichment across related traits (**Extended Data Fig. 6i,j**). These results indicate enrichment for genes linked to CHIP and malignancy risk among DVG. Finally, we performed PROGENy analysis^33^ to characterize the pathways most likely to be modulated by common and conserved ageing DVG which could contribute to oncogenic susceptibility. Top positively enriched pathways included MAPK, EGFR, TNF and NFkB. PI3K was the top negatively enriched pathway (Fig. 5k).

Together, these findings demonstrate that transcriptional variability in ageing HSC encodes clinically relevant programs linked to clonal selection in normal and malignant haematopoiesis, establishing DVG as a distinct and functionally meaningful axis of HSC state.

## Discussion

Ageing of the haematopoietic system is marked by clonal dominance, lineage bias and increased disease risk, yet the mechanisms by which these features emerge remain debated. It is unclear to what extent ageing reflects a global decline in stem cell function, stochastic drift, or selective amplification of specific stem cell subsets. Here we show that aging increases phenotypic variability in HSC pools, and this variation enables selection for HSC that are better adapted to the bone marrow niche as it changes across a lifespan. Importantly, the fate of an HSC clone is partially predictable both from its gene expression profile but also from its intra-clonal expression heterogeneity, revealing heterogeneity as an important axis regulating HSC fate. Aging associated DVG are not random but enriched for specific pathways involved in HSC fitness, and DVG in pre-transplantation HSC clones can predict clonal behaviour even two months post-transplantation. DVG predictive of clonal behaviour are enriched for genes involved in hematopoietic malignancy evolution and risk.

Early lineage-tracing studies established that HSC are intrinsically heterogeneous in fate and output, even under physiological conditions. Polylox-based clonal analyses using an artificial DNA recombination locus to identify clones, demonstrated that adult haematopoiesis is sustained by long-lived HSC clones exhibiting coherent, clone-specific lineage output patterns across haematopoietic compartments^34^. Subsequent integration of lineage tracing with transcriptomics revealed fate-associated molecular programs not apparent from reading out transcriptomic cell state alone^35^. Similarly, transposon-based tracing in unperturbed haematopoiesis showed that lineage bias and clonal contribution emerge upstream of classical progenitor boundaries^36^. Our results extend these observations into the ageing setting by demonstrating that while cell-intrinsic or cell-extrinsic ageing alterations do not substantially affect the overall clonal structure of HSC (per CellTag marked clones), individual HSC clones differ in their relative self-renewal and fitness in age-mismatched compared to age-matched transplantation settings.

Recent work has suggested that ageing-associated clonal expansions are not restricted to mutated clones, but instead represent a broader remodelling of the stem cell pool^6,11^. Epimutation-based lineage tracing in mouse and human haematopoiesis revealed that myeloid bias and reduced output in old age are confined to a subset of expanded clones, while many functionally young-like clones persist^11,37^. Our findings converge with this model by showing that aged environments preferentially expand better adapted clones, rather than uniformly impairing HSC function. Importantly, heterochronic transplantation reveals that identical sister clones diverge dramatically depending on host age, directly demonstrating that ageing niches impose selective pressures on intrinsic stem cell programs.

Reduced engraftment potential of HSC on a per cell basis is a well described phenomenon in ageing^38–40^ and is consistent with our observations (**Extended Data Fig. 5b**). At the same time, with ageing, the size of the HSC compartment increases. Our data suggest that HSC clonal expansion coupled with increased transcriptional heterogeneity might compensate for the loss of fitness of individual HSC, allowing increased adaptability to an altered microenvironment and preservation of haematopoiesis. At the same time, phenotypic variability can contribute to the increased risk of malignant transformation. Under these conditions, age-dependent effects manifest not as uniform shifts in clonal dominance, but as selective amplification of cells within clones whose pre-existing transcriptional states are compatible with the host environment. These findings support a model in which ageing primarily modulates the distribution of stem cell states and their selective responsiveness, rather than globally altering their capacity for clonal expansion. This framework further explains why early transcriptional variability retains predictive power for clonal outcomes, as selection operates on a pre-existing distribution of cellular states.

At the molecular level, ageing is associated with increased transcriptional variability and entropy within HSC, a feature often interpreted as regulatory noise. Our data suggest a complementary interpretation: increased variability may expose latent differences among clones, enabling selection in addition to random drift. This view aligns with studies of clonal competition under oncogenic stress, where pre-existing stem cell states dictate clonal responses to leukaemic mutations^19^. Together, these observations support a unifying framework in which intrinsic heterogeneity provides the substrate for both ageing-associated selection and malignant evolution.

The pathways enriched among conserved ageing DVG, including MAPK, EGFR, TNF and NFκB signalling, suggest potential mechanisms by which transcriptional variability may confer selective advantage in aged environments. Increased variation in TNF and NFκB pathway genes may enhance adaptability to the inflammatory signals characteristic of aged bone marrow niches, while variability in MAPK and EGFR-related programs could reflect heterogeneous responsiveness to intrinsic ageing-associated signals, both of which have established roles in HSC ageing^41,42^. Conversely, reduced variability in PI3K pathway genes, a known negative regulator of HSC self-renewal^43^, may reflect tighter constraint of quiescence programs in aged HSC. These observations are speculative and will require direct functional validation.

Finally, our ability to predict long-term clonal behaviour from early transcriptional states parallels findings in other systems where lineage history determines fate long before terminal differentiation. In haematopoiesis, early fate priming and clonal bias precede overt transcriptional divergence^44^, and clone-intrinsic programs such as the TCF15-associated self-renewal network govern long-term stem cell persistence^20^. By extending these principles to ageing, our work suggests that interventions targeting age-selected stem cell programs, rather than attempting global rejuvenation, may offer a more effective strategy to preserve tissue function and limit age-associated clonal disorders.

A previously underappreciated feature of this trajectory is its timing. To probe when across the human lifespan these variability programs first emerge, we reanalysed a scRNA-seq atlas of human bone marrow CD34+ HSPC spanning postconception week 10 through age 77^29^, restricting analysis to the HSC compartment. Within the dataset’s adult range, donors at 35–53 years (middle age) showed elevated mean-adjusted variance relative to young-adult donors at 17–32 years, with significant differences across young, middle-age and elderly (62–77 years) groups. The middle-age signal is thus established well before the typical onset of ageing-associated haematopoietic disorders including CHIP and leukaemia. Although cross-sectional and therefore not directly informative about within-individual progression, this observation nominates a candidate temporal window, potentially decades long, during which intra-clonal transcriptional variability could be monitored as an early indicator of clonal selection, before mutational drivers reach detectable variant allele frequency. Whether longitudinal measurement of variability signatures, alone or together with mutational profiling, can prospectively stratify CHIP-to-MDS/AML risk is an open question that the framework presented here makes tractable, and one that motivates prospective cohorts as a next step.

Several limitations of this study merit consideration. First, the Day 0 transcriptional baseline represents a post-expansion state following 15 days of *ex vivo* culture; while the unmanipulated HSC dataset independently recapitulates the ageing heterogeneity signal, the predictive analyses are necessarily confined to this experimental system. Second, all key conclusions derive from transplantation-based assays; the extent to which these dynamics operate in native, unperturbed haematopoiesis remains to be established. Third, cross-validation for the predictive models was performed at the cell level; clone-level cross-validation was not feasible given the limited number of clones, and performance estimates may therefore reflect some degree of intra-clone structure. Finally, while transcriptional variability correlates with and predicts clonal fate, the causal role of heterogeneity as a driver – rather than a marker – of selective advantage has not been directly demonstrated and will require future perturbation studies.

## Methods

### Experimental design

This study tests whether pre-existing transcriptional heterogeneity in wild-type haematopoietic stem cells (HSC) predicts clonal behaviour during ageing and across young versus aged haematopoietic environments. We combined CellTag-based clonal lineage tracing, single-cell RNA sequencing (scRNA-seq), and homo- and heterochronic transplantation assays to longitudinally track sister HSC clones and quantify self-renewal, clonal fitness, differentiation output, and intra-clonal heterogeneity.

All molecular and functional assays were performed on matched cohorts of mice, and Day 0 pre-transplant transcriptional states were explicitly linked to post-transplant clonal outcomes measured at a later endpoint. No mutant models, chromatin conformation assays, or epigenomic profiling were included in this study.

### Mice

Young C57BL/6j female mice (2 months old) and old C57BL/6j female mice (20–22 months old) were obtained from the National Institute of Ageing repository. Mice were acclimatized for 2–4 weeks at the University of Colorado Anschutz Medical Campus Laboratory Animal Shared Resources before being used for experiments. Mice were housed in specific pathogen-free animal facilities, maintained at 21 °C (±1 °C) and 35% humidity with a 14 h:10 h light:dark cycle (light 06:00–20:00). Animal protocols were approved by the CU Anschutz Institutional Animal Care and Use Committee in accordance with the NIH Guidelines for the Care and Use of Laboratory Animals.

### HSC isolation and purification

Mice were sacrificed by CO_2_ asphyxiation followed by cervical dislocation, and immediately placed on ice. Mouse carcasses were disinfected with ethanol and the tissues processed in a sterile environment in a biosafety cabinet. Bones (femurs, tibia, iliac crests and spine) were removed with a scalpel, placed in a sterile mortar containing 15 mL of FACS buffer (PBS, 2% fetal bovine serum, 2 mM EDTA). The suspension of bone marrow cells was passed through a 70 μm cell strainer, centrifuged at 500 g for 5 minutes at 4 °C. Red blood cells were hemolyzed with ammonium-chloride-potassium (ACK) lysis buffer (8 g L^−1^ ammonium chloride, 1g L^−1^ potassium hydrogen carbonate, 0.2 mM EDTA). To remove dead cells and debris cells were centrifuged on top of a layer of Histopaque 1119 and the upper layer was collected. Cells were stained with CD117 magnetic microbeads (Miltenyi) and CD117^+^ cells were enriched by magnetic separation on LS columns. HSC were sorted with the MoFlo XDP100 or the Sony MA900 cell sorter based on the following cell surface markers: lineage^NEG^ (Ter119, CD3e, CD8, B220, CD11b, Gr-1), Sca-1^+^, c-Kit^+^, CD48^NEG^, and CD150^+^.

### Lentiviral plasmid library preparation and lentivirus production

The CellTag V1 plasmid library expressing EGFP was obtained from Addgene (#115643). 50 ng of CellTag V1 plasmid DNA was diluted in 50 μL with water and transformed in 10 tubes of Stellar chemically competent cells (Takara cat# 636763). An aliquot of bacteria was plated on a 10 cm LB-Agar carbenicillin plate at multiple serial dilutions to ensure a >100-fold colonies/celltags representation. Bacteria were expanded in terrific broth with 100 μg mL^−1^ carbenicillin and DNA was isolated with Qiagen Plasmid Plus Maxi Kits (#12963). For lentiviral production HEK293T cells were cultured in DMEM high glucose, pyruvate, supplemented with 10% FBS, 1% Pen/Strep in 10 cm dishes at 37 °C, 5 % CO2 to 50–70% confluence. The medium was changed 2 hours to the transfection and 15 μM chloroquine was added. A transfection mix was prepared by combining the lentiviral plasmids: Celltag V1 (10 μg/plate), psPAX2 (10 μg/plate), pMDG2.G (6 μg/plate) with PEI Max (at a 1:3 mass ratio) in OptiMEM medium (1 mL per 10 cm plate). The mix was shaken by vortexing for 30 seconds, incubated for 15 minutes at room temperature and then added drop by drop to the HEK293T cells and mixed by swirling. The medium was changed at 24 hours after the transfection and collected 24 and 48 hours later. The collected medium was filtered (0.45 μm) aliquoted in high-speed PPCO centrifuge tubes. A layer of 5 mL sterile sucrose-TNE solution (20% (w/v) sucrose, 50 mM Tris-HCl, pH 7.4, 100 mM NaCl, 0.5 mM EDTA) was gently deposited at the bottom of the tube, then tubes were centrifuged at 40,000 g for 2 hours at 4 °C. The supernatant was carefully aspirated, the pellet was resuspended in Polyvinyl alcohol (PVA) medium (HAM-F12 medium supplemented with 100 U/mL Penicillin/Streptomycin, 2mM L-Glutamine, 10 mM HEPES, 1X Insulin, Transferrin, Selenium, Ethanolamine, 0.1% PVA) and stored in aliquots at −80 °C.

### CellTag barcoding of HSC

Purified HSC cells were plated on 96-well fibronectin coated plates in HAM-F12 medium supplemented with 100 U mL^−1^ Penicillin/Streptomycin, 2 mM L-Glutamine, 10 mM HEPES, 1X Insulin, Transferrin, Selenium, Ethanolamine, 0.1% PVA, 10 ng/mL recombinant mouse SCF and 100 ng/mL recombinant mouse TPO. The Celltag V1 lentivirus was titrated directly on young HSPC and was used for experiments at a dilution achieving 70–90% transduction efficiency. Transduction of HSC was done by plating ~10,000 cells/well with the appropriate dilution of Celltag V1 lentivirus in freshly coated fibronectin coated wells. 100 μg/mL Synperonic F-108 (Millipore-Sigma #07579–250G-F) was added to increase the transduction efficiency. Medium was changed every 2–3 days and fibronectin plates were replaced every week. Cells were cultured for 15 days after lentiviral transduction, at which point an aliquot of cells was collected, EGFP^+^ HSPC cells (lineage^NEG^ (Ter119, CD3e, CD8, B220, CD11b, Gr-1), Sca-1^+^, c-Kit^+^) and as indicated a fraction of more differentiated cell populations were sorted and captured for Day 0 pre-transplant analysis, the remainder of the cells were transplanted into pre-conditioned recipient mice (see below).

### Flow cytometry

For quality control a portion of cultured cells were resuspended in FACS buffer with antibodies and 50 μg mL^−1^ normal rat IgG to block non-specific signals. Cells were incubated for 30 minutes in the dark at 4 °C. Cells were washed once and analysed with a CytoFLEX S flow cytometer (BD). HSC in vitro were defined as lineage^NEG^ (Ter119, CD3e, CD8, B220, CD11b, Gr-1), Sca-1^+^, c-Kit^+^, EPCR^+^.

For *Procr* validation, ten individual HSC samples from old or young mice were transduced with an empty-vector control lentiviral vector expressing dTomato and cultured for 15 days. Variability in the expression of PROCR/EPCR was defined by a coefficient of variation (Modified CV) obtained by dividing the robust standard deviation by the geometric mean for each sample calculated by using the FlowJo software.

### Transplantation assays

Busulfan was dissolved in DMSO at 20 mg mL^−1^, at the moment of administration, the busulfan solution was diluted to 2 mg mL^−1^ with pre-warmed sterile saline solution (0.9 g L^−1^ NaCl in water) and kept warm (~60 °C). Mice were injected intraperitoneally with 10 μL g^−1^ of body weight with the diluted busulfan 4 days before the transplantation. Anti-CD4 (BioXcell clone GK1.5) and anti-CD8 antibodies (BioXcell, clone 2.43) were diluted in sterile PBS and mice were administered 30 μg of each antibody in 200 μL by intraperitoneal injection 2 days before the transplantation. Cells were detached from fibronectin plates by pipetting. Cells were centrifuged at 500 g for 5 minutes at room temperature, resuspended in sterile PBS and 200 μL of cell suspension was injected into the tail vein of conditioned recipient mice.

In vitro expanded, barcoded cells were analysed by flow cytometry for quality control to monitor the fraction of HSC/HSPC and the EGFP positivity.

For homo-chronic transplantation (young donors into young recipients; old donors into old recipients) HSC from young and old mice (n = 3 each) were individually lentivirally barcoded, expanded, individually captured for scRNA-seq and transplanted into different recipient age-matched mice.

For homo/hetero-chronic transplantation HSC from young and old mice (n = 3 each) were individually barcoded and expanded. For scRNA-seq and transplant, the three young replicates and the three old replicates were pooled into young and old batches of cells. Cells were injected into mice of the same age (YY, OO: homo-chronic transplants) or different ages (YO, OY: heterochronic transplantation).

For scRNAseq, cells for individual donor mice were distinguished by staining each replicate with one of three Totalseq B hashing antibodies (Biolegend #B0301, #B0302, #B0303) prior to pooling.

At the experimental endpoint, bone marrow cells were harvested from recipient mice as described above. HSC enriched EGFP+ cells were sorted and submitted for capture and scRNA-seq.

### Single-cell RNA sequencing

Single-cell RNA-seq libraries were generated using the Chromium platform (10x Genomics). For each sample, at least 10,000 were loaded for target capture of 10,000–20,000 cells with the Chromium GEM-X Single cell 3’ Chip Kit v4, cat# 1000690 and libraries were produced with the GEM-X Universal 3’ Gene Expression v4, cat# 1000691. Libraries were sequenced with the NovaSeq S to a depth sufficient to recover both transcriptomes, CellTag barcodes and when present antibody hashes.

### scRNA-seq preprocessing and cell type annotation

Raw sequencing data were processed using Cell Ranger (v9.0.1)^45^. Multiplexed hashtag-labelled single-cell RNA-seq data comprising three replicates were extracted using the Cell Ranger multi command. Cells with fewer than 200 detected genes were excluded. Downstream normalization, integration, clustering, and visualization were performed using Seurat^46^.

Highly variable genes (HVGs), defined as genes exhibiting high cell-to-cell variation relative to their average expression level, were first identified to capture the most informative features of the dataset. Dimensionality reduction was carried out using principal component analysis (PCA; n=30 PCs). Batch effects were corrected with Harmony^47^ (θ=1), followed by UMAP for visualization. Cells were then embedded using Seurat’s FindNeighbors and clustered using FindClusters (resolution=0.8). Cell types were annotated based on canonical haematopoietic marker gene expression. HSC and progenitor populations were defined *in silico* and used for all downstream clonal analyses.

### CellTag barcode recovery and clone calling

CellTag barcodes were extracted from scRNA-seq reads and summarized as a CellTag UMI count matrix using the CellTagR workflow (https://github.com/morris-lab/CellTagR). To determine CellTag presence per cell, CellTags supported by ≥ 2 UMIs in a given cell were retained, and lower-count CellTags were treated as absent. Clone calling was then performed by computing pairwise Jaccard similarities between cells using the filtered and binarized CellTag count matrix (after metric filtering by CellTags per cell ≥1 and ≤ 20). Cells were grouped into clones based on the resulting Jaccard similarity matrix, and a clone was retained when within-clone Jaccard similarity ≥ 0.7.

### Definition of sister clones

Sister clones were defined as clones detected at both baseline (Day 0, pre-transplant) and the post-transplant endpoint. Only such shared clones were included in longitudinal analyses of clonal self-renewal, fitness, and transcriptional heterogeneity.

### Clonal self-renewal analysis

Clonal self-renewal was quantified using output activity: Aᵢ = (f_non-HSC,i + 10^−4^) / (f_HSC,i + 10^−4^)

where f_HSC,i and f_non-HSC,i denote the frequency of clone *i* in HSC and non-HSC compartments, respectively. Clones with Aᵢ <1 were classified as self-renewing.

Analyses were restricted to clones:

1. detected at both Day 0 and post-transplant.
2. with combined counts ≥ 5 within the corresponding donor group (OO + OY for old donors; YO + YY for young donors).
3. with HSC frequency > 0.003 at the post-transplant timepoint (applied for Fig. 5a).

### Clonal fitness index

Clonal fitness was defined as the log_2_ fold-change in normalized clone size between Day 0 (pre-transplantation) and the post-transplant endpoint. Only clones detected at both timepoints and meeting size thresholds were included. Clone sizes were normalized to total cell numbers per sample to enable comparisons across experiments.

### Clonal size stratification

Clone size was defined as the number of cells per clone at a given timepoint. For descriptive analyses, clones were stratified by relative frequency as:

1. Small (<0.01)
2. Medium (0.01–0.05)
3. Large (>0.05)

### Assessment of transcriptional heterogeneity

#### Intra-clonal heterogeneity

Intra-clonal transcriptional heterogeneity was quantified using clone-level gene expression profiles. For each clone, average gene expression was calculated across all cells belonging to the same clone. Analyses were restricted to predefined gene sets, including differentially variable genes. For each group of clones (for example, high- and low-fitness groups), the mean expression of each gene was calculated across clones within the group. A global reference was defined as the mean expression across all clones. For each gene, the deviation of group-level expression from the global mean was computed. The absolute deviation values were used as a proxy for intra-clonal transcriptional heterogeneity, capturing the extent to which group-specific transcriptional programs diverge from the overall population.

### Entropy-based heterogeneity

Shannon entropy was calculated from the distribution of cell states within each clone and normalized by clone size where appropriate. Only clones containing ≥2–5 cells were included to ensure statistical robustness.

### Differential expression and gene set enrichment

Differential gene expression analyses were performed using MAST^48^ on the top 2,000 highly variable genes. Gene set enrichment analyses were conducted to identify transcriptional programs associated with clonal fitness, self-renewal, and ageing-associated outcomes. PROGENy was applied to test for pathway enrichment and MAGMA (multi-marker analysis of genomic annotation) gene set analyses were performed on clonal-haematopoiesis related GWAS.

### PCA and group separation.

For visualization (Fig. 4f), PCA was computed on the integrated single-cell expression matrix using variable features; 2D kernel density contours and group density peaks (KDE modes) are shown on PC1-PC2. For statistical inference (Extended Data Fig. 4j), expression was aggregated to pseudo-bulk profiles per mouse (n = 12; Seurat::AverageExpression). Pseudo-bulk profiles were mapped onto the single-cell HSC reference PCA (PC1–PC30) using Seurat FindTransferAnchors and IntegrateEmbeddings. PERMANOVA (vegan::adonis2) on Euclidean distances from PC1–PC30 (9,999 permutations) tested global group separation and donor × host-age interaction (distance ~ donor_age * host_age). Pairwise comparisons used Benjamini–Hochberg correction. Multivariate dispersion was assessed by PERMDISP (vegan::betadisper) using the same PC1–PC30 distances. P values below 1/(m+1) are reported as upper bounds.

### Single-cell pathway analysis

single-cell pathway enrichment analysis was performed using SCPA v1.6.2 (https://github.com/jackbibby1/SCPA/)^49^, with Hallmark gene sets (MSigDB category H) from the Molecular Signatures Database (MSigDB). Pathway activity was assessed at the single-cell level, and gene sets with a −log_10_(q-value) > 2 and an adjusted p-value < 0.01 were considered significantly enriched.

### Machine learning-based prediction of clonal behaviour

Supervised machine learning models were implemented using a random forest classifier to predict Day 60 self-renewal outcomes as labels from both Day 0 transcriptional states (early prediction) and Day 60 transcriptional states (late prediction).

The model input consisted of single-cell gene expression profiles, with each cell represented by its gene expression features, and the corresponding Day 60 self-renewal outcomes as labels.

Model performance was evaluated using five-fold cross validation. In each fold, the dataset was randomly partitioned into training and validation subsets to ensure robustness and reduce overfitting.

The feature sets included low-output marker genes and aging-up DVGs. Aging-up DVGs were identified independently in three datasets using log2 fold-change cutoffs of 0 and 0.1, and only genes with significant differential variability (FDR-adjusted p value < 0.05) were retained. For each cutoff, genes identified in at least two of the three datasets were selected to generate a robust DVG gene set. These DVG gene sets, together with low-output marker genes, were used as input for a random forest model. Features were ranked according to importance scores, and the top-ranked genes were used for downstream analyses.

All analyses were restricted to HSC and MPP cell populations.

### TCGA-LAML survival plot

The TCGA acute myeloid leukaemia (LAML) cohort was used to evaluate the clinical relevance of gene signatures derived from machine learning models. Gene expression and corresponding clinical data were obtained from The Cancer Genome Atlas (TCGA).

For each sample, a signature score was calculated based on the expression of genes selected from the machine learning models. Patients in the TCGA-LAML cohort were stratified into high- and low-expression groups according to the median signature score.

Overall survival (OS) was assessed using Kaplan–Meier analysis based on OS time and status. Statistical significance was determined using the log-rank test. Hazard ratios were estimated using Cox proportional hazards regression models.

All analyses were restricted to HSC and MPP cell populations.

### Statistical analysis

All statistical analyses were performed using R and Python. Multiple testing correction was conducted using the Benjamini–Hochberg procedure unless otherwise stated. Two-tailed Wilcoxon rank-sum tests were used for two-group comparisons unless otherwise specified.

### Human lifespan HSC reanalysis

To assess when across the human lifespan the ageing-associated variability signature first emerges, we reanalyzed the publicly available scRNA-seq atlas of human bone marrow CD34+ haematopoietic stem and progenitor cells (HSPCs) spanning postconception week 10 through age 77 (GEO accession GSE189161; raw data dbGaP phs002750)^29^. We restricted analysis to the HSC compartment and binned postnatal donors as young adults (25, 25, 32 years; n = 3 donors), mid-age (35, 45, 53 years; n = 3 donors) and elderly (62, 76, 77 years; n = 3 donors). Mean-adjusted variance was computed using the same scCloneVar pipeline applied to mouse HSC, treating donors as biological replicates. Group differences across young, mid-age and elderly were tested by Wilcoxon rank-sum on per-gene mean-adjusted variance scores. Differential variability gene (DVG) sets were derived using the same statistical thresholds as for the mouse HSC analyses.

## Supplementary Files

This is a list of supplementary files associated with this preprint. Click to download.

- CelltagExtendedDataTables060426.xlsx
- CellTagExtendedData060526Final.pdf

## Data Availability

The scRNA-seq data generated in this study have been deposited in the Gene Expression Omnibus (GEO) under the following accession numbers: Unmanipulated scRNA-seq: GSE325995. Celltag scRNAseq experiment 1: GSE327449. Celltag and hashtag scRNA-seq experiment 2: GSE326529. Source code availability: https://github.com/LabShengLi/CellTag-Main-Figure-code scCloneVar R package code availability: https://github.com/LabShengLi/scCloneVar External human scRNA-seq datasets are publicly available in the Gene Expression Omnibus (accessions GSE180298 and GSE189161). TCGA Acute Myeloid Laeukemia (LAML) data were accessed through The Cancer Genome Atlas (TCGA) Genomic Data Commons (GDC) Data Portal.
